# A Case of Green Nail Syndrome Diagnosed in the Emergency Department

**DOI:** 10.7759/cureus.57032

**Published:** 2024-03-27

**Authors:** Philip Carhart, James Espinosa, Alan Lucerna

**Affiliations:** 1 Emergency Medicine, Jefferson Health, Stratford, USA

**Keywords:** fingernails and physical diagnosis, emergency department evaluation of fingernail abnormalities, fingernail abnormailities, goldman-fox syndrome, pseudomonal green nail syndrome, green nail syndrome

## Abstract

Green nail syndrome (GNS) is a rare diagnosis in which a patient presents with green-yellow, green-blue, or green-brown discoloration of a finger or toenail. It occurs due to a *Pseudomonas aeruginosa* infection of the nail. *Pseudomonas aeruginosa* produces pigments that can infuse into the underside of the nail plate, creating a color change. Here, we present the case of a 34-year-old female with a green-brown area of discoloration of her right middle finger in which the diagnosis of GNS was made. The patient used acrylic nails, which is a known risk factor. The characteristic clinical context and physical exam findings of green-yellow, green-blue, or green-brown nail discoloration are said to be sufficient to make a working diagnosis of GNS. The differential diagnosis of GNS includes a subungual hematoma, a subungual melanoma, and exogenous yellow pigment exposure. The history, physical examination, and response to treatment will help to clarify the differential. Ciprofloxacin is a commonly used empiric treatment. Laboratory testing of a nail clipping can be used in cases that do not respond to treatment. Cultures of nail clippings appear to be specific, but not sensitive, to the detection of *P. aeruginosa.* Our patient saw an immediate improvement within a week of treatment, with complete resolution in eight weeks. This is a typical timeframe. Knowledge of the syndrome can be helpful to reduce patient anxiety and guide effective therapy.

## Introduction

*Pseudomonas aeruginosa* infection of a nail is the most common pathogen associated with bacterial infection of the nail [1]. The uniqueness of a *P. aeruginosa* infection of the nail is that it produces two pigments (pyoverdine and pyocyanin), which can produce a green-yellow, green-blue, or green-brown discoloration of the affected nail, known as green nail syndrome (GNS). In addition to *P. aeruginosa*, other bacteria have been identified that can cause nail discoloration. Culture-negative results have occurred as well, probably because cultures did not grow the causative organism [2-4]. These pigments diffuse into the underside of the nail plate [5]. Goldman and Fox published the first report on GNS, in which they linked the syndrome to *P. aeruginosa*, and therefore GNS is sometimes referred to as Goldman-Fox syndrome [2]. This phenomenon is considered to be rare [1,3].

## Case presentation

**Figure 1:**
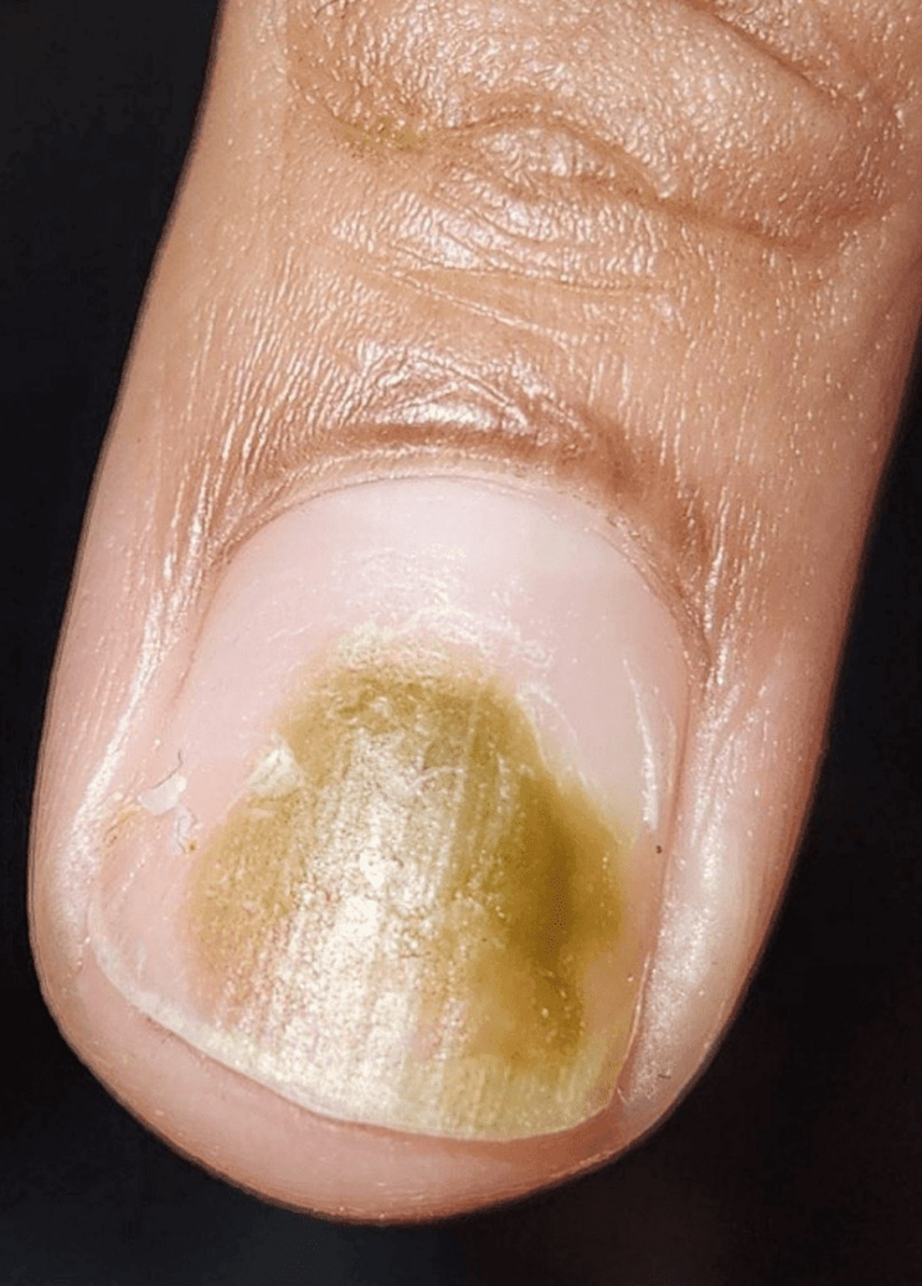
Patient's fingernail on presentation showing green-brown discoloration

A 34-year-old female patient presented to the ED with a complaint of green discoloration on her right middle fingernail for a one-week duration. She had been using acrylic nails for several months. The current set of acrylic nails had been placed two weeks before the ED presentation. She first noticed the area of discoloration after she accidentally struck the acrylic nail on a household object, which loosened the acrylic nail. She removed the acrylic nail several days later and noticed the green color of the underlying fingernail. She denied pain, swelling, redness, or warmth of the finger. She denied fevers or chills. Her past medical history included intermittent asthma. On physical exam, there was a distinct green-brown discoloration of the nail plate on the right middle finger (Figure 1).

The nail plate was firmly attached to the nail bed. The examination of the affected digit, hand, and arm was otherwise normal, with no tenderness, warmth, or lymphangitic streaking. The image of the nail was reviewed remotely by a dermatologist, who agreed with the ED's impression of GNS. The patient was treated with ciprofloxacin 500 mg twice per day for one week. The patient stated that she noted an initial immediate improvement in the discoloration of the nail as well as a mild shrinkage in the size of the infected area within one week of the initiation of antibiotics. She followed up with her primary doctor as advised, who recommended avoidance of acrylic nail placement and manicures. At the two-month follow-up by telephone, the patient stated that the color change had completely resolved.

## Discussion

*Pseudomonas aeruginosa* is widespread and can be found in water, soil, plants, animals as well as in humans [1]. It can cause infections on a local and systemic level [2]. However, it is not a part of the normal flora of healthy, dry skin [2]. Chronic exposure to water is a known risk factor for GNS. It has been described in swimmers, dishwashers, and bakers [1] as well as in plumbers [2]. Chronic nail pathology, such as psoriasis involving the nail, can be a risk factor [1]. Patients with HIV infection may be more vulnerable to GNS [5]. Diabetes mellitus can also be a risk factor for GNS [6]. Repeated microtrauma to a finger or toe, or a single episode of direct trauma, can be risk factors [1,6]. Pre-existing fungal disease of the nail has been associated with GNS [7]. Finger trauma has been associated with GNS in which *Citrobacter braakii* was the causative organism [8]. The patient used acrylic nails. Acrylic nail use for more than one month has been associated with GNS [9]. It is felt that the use of acrylic nails can lead to the entrapment of water between the artificial nail and the natural nail, leading to infection [9]. It is noteworthy that artificial nail use in healthcare nurses has been linked to nosocomial infections [10].

The most common presentation is in a single nail of the hand or foot, most commonly the thumb and great toe [1]. The characteristic discoloration of the nail can be accompanied by a paronychial infection at the base or side of the nail or even by onycholysis of the distal nail. The patient here did not have paronychial involvement and the nail was tightly attached to the nailbed. The characteristic green-yellow, green-blue, or green-brown nail discoloration distinguished during a physical examination is sufficient for a working diagnosis of GNS [1,3]. The presence of a subungual hematoma, subungual melanoma, and exogenous yellow pigment exposure make for the differential diagnosis of GNS. Both patient history and physical examination help clarify the differential [1].

Laboratory testing of a nail clipping can be used in cases that do not respond to treatment, however, empiric treatment can be initiated first [1]. While cultures of the nail clippings can appear to be specific about *Pseudomonas* they are not sensitive. A retrospective review of 26 cases of GNS found that over 80% of the patients achieved complete resolution with anti-pseudomonal therapy but only 42% tested positive for* Pseudomonas* [1].

A wide variety of treatment options exist. If present, elements of the nail showing onycholysis can be removed. After removal, treatment with compresses of 1% acetic acid has been described [1]. However, acetic acid can irritate adjacent skin [11]. Topical treatment with gentamycin or tobramycin eyedrops has been described in case reports, especially where onycholysis has occurred, thus allowing drops to penetrate under the nail [1,11]. Oral therapy with ciprofloxacin has been described [3,4] and has been shown to have good clinical effectiveness in GNS [9]. A trial of oral fluoroquinolone can prevent unnecessary laboratory studies [12] and was the approach used in this case. Our patient showed significant improvement in one week.

Even with successful treatment, evidenced by a progressive reduction in the size of the discoloration, complete resolution of the color change can take several months [1]. Resolution of the nail discoloration required two months in the case of our patient. If the discoloration does not resolve, a culture of nail cuttings can be performed. Sometimes the total removal of the nail is needed [1].

## Conclusions

The patient presented in this case illustrates several important points concerning GNS. First, the discoloration of the nail in GNS is characteristically blue-green, yellow-green, or green-brown. Second, a brief trial of empiric oral therapy with a fluoroquinolone, such as ciprofloxacin, should lead to resolution within eight weeks. Third, if there is no resolution within that time frame, cultures of nail clippings can be obtained. Failure to resolve raises concern for subungual melanoma. Fourth, awareness of GNS can help guide effective therapy and reduce patient anxiety.
